# Thermal ablation as an alternative to liver transplantation for hepatocellular carcinoma with clinically significant portal hypertension: propensity score matching study

**DOI:** 10.3389/fonc.2023.1103347

**Published:** 2023-06-26

**Authors:** Yinglin Long, Zhou Yang, Qingjing Zeng, Zhongqi Liu, Erjiao Xu, Xuqi He, Lianxiong Yuan, Binsheng Fu, Kai Li

**Affiliations:** ^1^ Department of Ultrasound, Guangdong Key Laboratory of Liver Disease Research, the Third Affiliated Hospital of Sun Yat-sen University, Guangzhou, China; ^2^ Department of Hepatic Surgery and Liver Transplantation Center of the Third Affiliated Hospital, Organ Transplantation Institute, Sun Yat-sen University, Organ Transplantation, Research Center of Guangdong Province, Guangzhou, China; ^3^ Department of Anesthesiology, Sun Yat-sen Memorial Hospital, Sun Yat-sen University, Guangzhou, China; ^4^ Department of Science and Research, Guangdong Key Laboratory of Liver Disease Research, the Third Affiliated Hospital of Sun Yat-sen University, Guangzhou, China

**Keywords:** thermal ablation, liver transplantation, hepatocellular carcinoma, portal hypertension, propensity score matching

## Abstract

**Purpose:**

The objectives were to investigate the safety and efficacy of thermal ablation as an alternative to liver transplantation for hepatocellular carcinoma patients with clinically significant portal hypertension (CSPH).

**Materials and Methods:**

From July 2016 to September 2019, hepatocellular carcinoma patients with CSPH treated by liver transplantation (N=37) or thermal ablation (N=114) were enrolled. Cumulative intrahepatic recurrence, overall survival and major complications were compared by propensity score matching.

**Results:**

In the two matched groups, the 1-, 2-, and 3-year intrahepatic recurrence rates for the ablation group (22.3%, 50.0%, and 50.0%, respectively) were significantly higher than those for the transplantation group (4.5%, 4.5%, and 4.5%, respectively) (P=0.016). The 1-, 2-, and 3-year overall survival rates were comparable between the two groups [96.1%, 88.7%, and 88.7%, respectively (ablation group) *vs*. 84.6%, 76.2%, and 76.2%, respectively (transplantation group)] (P=0.07). The major complication rate for the ablation group [4.8% (3/62)] was significantly lower than that for the transplantation group [36.0% (9/25)] (P<0.001).

**Conclusions:**

Thermal ablation is a safe and effective alternative for hepatocellular carcinoma patients with CSPH.

## 1 Introduction

Hepatocellular carcinoma (HCC) is the second leading cause of cancer-related mortality worldwide (1). As previously reported, liver cirrhosis caused by various factors is an important risk factor for the occurrence and development of HCC (2). Therefore, HCC mostly develops on a background of liver cirrhosis and portal hypertension. Generally, portal hypertension is identified either instrumentally by a hepatic venous pressure gradient measurement ≥10 mmHg or clinically by the presence of esophageal and gastric varices and/or a platelet (PLT) count <100*10^9^/L associated with splenomegaly (3–5). Based on this concept, the PLT count is influenced by the severity of portal hypertension, and thrombocytopenia remains an important factor of morbidity and mortality for HCC patients with portal hypertension.

According to the current guidelines, liver transplantation (LT) is a favorable treatment option for early-stage HCC patients with cirrhosis and portal hypertension because it can remove the tumor as well as treat the underlying cirrhosis (2). However, the scarcity of donor organs, high cost of surgery and relatively high risk of posttransplantation complications (6, 7) have greatly limited the general application of LT. Therefore, it is reasonable to seek an effective and safe alternative for these patients.

According to current opinions, surgical resection is not recommended for HCC patients with cirrhosis and portal hypertension (2, 8). Locoregional ablative therapy, on the other hand, may be more suitable for early-stage HCC patients with cirrhosis (2, 9, 10). However, significant portal hypertension is also considered a relative contraindication for thermal ablation (TA) in practice. One recent study (11) demonstrated that the presence of clinically significant portal hypertension (CSPH) did not independently increase the risk for radiofrequency ablation (RFA). Moreover, contrast-enhanced ultrasound (CEUS) was possibly useful for monitoring intraprocedural bleeding in patients with abnormal coagulation functions during ablation (12). However, since the previous reports were one-arm studies, there is insufficient evidence on the clinical value of TA in terms of therapeutic effect and safety for HCC patients with CSPH.

Therefore, this retrospective study investigated the safety and efficacy of TA as an alternative to LT for HCC patients with CSPH through propensity score matching.

## 2 Methods

### 2.1 Inclusion and exclusion criteria

This retrospective nonblinding study was approved by the Institutional Review Board of our hospital with the registration number of [2020]02-029-01. Written informed consent was waived.

From July 2016 to September 2019, patients with HCC were retrospectively enrolled. The inclusion criteria were as follows: (1) clinically or pathologically diagnosed with HCC; (2) having undergone LT or TA at our hospital; (3) no more than 3 tumors with a maximum diameter of no more than 50 mm; and (4) identification and characterization of CSPH by the presence of esophageal and gastric varices and/or a PLT count <100 * 10^9^/L, which is associated with splenomegaly.

The presence of esophageal and gastric varices was assessed by means of upper-digestive endoscopy and was classified as absent, small, medium or large. For the purpose of this study, we present this variable as the absence or presence of varices. Splenomegaly was defined as a spleen length ≥11 cm.

Patients lost to follow-up within 3 months after LT or TA procedures were excluded from this study.

Among the enrolled patients, patients who underwent LT were grouped into the LT group, while those who underwent TA were grouped into the TA group. The application of TA or LT was decided by the patients after the doctor explained the recommendations from multidisciplinary experts, risks and benefits, and complications and prognoses of the currently available treatment modalities.

A total of 151 patients (223 lesions) were enrolled in this retrospective study. No patients were removed during the follow-up period. Among them, 114 patients (99 males and 15 females, aged 54 ± 11 years old) with 157 lesions were grouped into the TA group, while 37 patients (30 males and 7 females, aged 53 ± 11 years old) with 66 lesions were grouped into the LT group.

The baseline characteristics of the TA and LT groups are presented in **Table 1**. Significant differences between the two groups were observed in the following parameters: primary or recurrent tumor, single or multiple tumor and maximum tumor size. The median follow-up time was 14 (3-36) months for the TA group and 17 (3-43) months for the LT group.

**Table 1:** Demographic and clinical characteristics of all enrolled patients before and after propensity score matching.

	Before propensity score matching			After propensity score matching			
Characteristic	TA (N=114)	LT (N=37)	*P*	TA (N=62)		LT (N=25)	*P*
**Sex (male/female)**	99/15	30/7	0.388	53/9		21/4	1.000
Age* (years)	54 ± 11	53 ± 11	0.498	55 ± 12		54 ± 10	0.751
**BMI (≤18.5/>18.5)**	6/108	5/32	0.093	3/59		4/21	0.101
**Cause of cirrhosis (HBV/HCV/alcohol/drug)**	98/10/5/1	33/2/0/2	0.176	53/5/3/1		23/1/0/1	0.539
**History of GI bleeding (Yes/no)**	32/82	5/32	0.074	18/44		3/22	0.106
**Primary/recurrent tumor**	78/36	17/20	0.014	38/24		14/11	0.649
PLT (≤50*10^9/L/>50*10^9/L)	80/34	30/7	0.195	48/14		18/7	0.593
**PT (≤14.5 s/>14.5 s)**	4/110	0/37	0.572	61/1		25/0	1.000
ALB* (≤ 35 g/L/>35 g/L)	47/67	21/16	0.099	30/32		13/12	0.760
**TB (≤17.1 µmol/L/>17.1 µmol/L)**	34/80	12/25	0.765	19/43		8/17	0.902
**CR (µmol/L)**	73 (21-123)	73 (40-236)	0.892	75 ± 18		81 ± 40	0.346
**AFP (≤400 ng/mL/>400 ng/mL)**	103/11	32/5	0.507	54/8		22/3	1.000
**CHILD-PUGH (A/B)**	68/46	15/22	0.042	41/21		11/14	0.057
**Single/multiple**	82/32	18/19	0.009	34/28		15/10	0.660
**Maximum tumor size (≤30 mm/>30 mm)**	96/18	24/13	0.011	45/17		16/9	0.429
**Tumor number**	N=157	N=66		N=99		N=39	
**Tumor location (segment 1/2/3/4/5/6/7/8)**	0/14/9/17/27/36/31/23	1/11/1/4/14/12/11/12	0.230	0/9/3/12/15/20/22/18		1/7/1/2/7/8/4/9/	0.298

TA, thermal ablation; LT, liver transplantation; BMI, body mass index; HBV, hepatitis B virus; HCV, hepatitis C virus; GI, gastrointestinal; PLT, platelet; PT, prothrombin time; INR, international normalized ratio; ALB, albumin; TB, total bilirubin; CR, creatinine; AFP, alpha-fetoprotein.

*The distribution of age and ALB satisfied the normal distribution.

### 2.2 Equipment

#### 2.2.1 Liver transplantation equipment

Routine surgical instruments were used during the LT operation.

#### 2.2.2 Ultrasound equipment and contrast agent

MyLab Twice and MyLab Class US machines (Esaote, Genoa, Italy) with the convex probe CA541 (frequency: 1-8 MHz) or CA431 (frequency: 4-10 MHz) were employed for image guidance of electrode or antenna placement. SonoVue (Bracco, Milan, Italy) was injected *via* the antecubital vein or central vein, followed by 5 ml of saline. When necessary, SonoVue was injected repeatedly.

#### 2.2.3 Ablation equipment

RFA and microwave ablation (MWA) were used in the present study. RFA was performed with a cooled-tip RFA system (Covidien, Mansfield, MA, USA), and MWA was performed with an internally cooled microwave antenna (Kangyou Corp., Nanjing, China) and a microwave generator (Kangyou Corp., Nanjing, China) of 2450 MHz.

### 2.3 Preoperative treatment

Before LT or TA, serum albumin was administered intravenously to correct hypoproteinemia. The PLT level and prothrombin time (PT) were monitored. Anti-PLT or anticoagulation drugs were withdrawn for at least 7 days before the operation. Massive ascites was corrected by tube drainage and serum albumin supplementation.

### 2.4 Liver transplantation

Classic or piggyback orthotopic LT was performed under intratracheal general anesthesia. During the transplantation operation, the diseased liver was excised, the vena cava was anastomosed, and the supply of the donor liver was recovered. Then, the portal vein, hepatic artery and biliary tract were anastomosed. For the enrolled patients, the total blood loss was 1000 (500-8000) ml on average. The no-liver period was 46 (25-65) minutes per person. The cold ischemia period was 6.5 (5.2-8.0) hours per person.

### 2.5 Local thermal ablation

The TA procedure was performed by four specialists with more than 5 years of experience in ablation procedures. The ablation modality, auxiliary procedure and puncture path were discussed before the ablation procedure by at least two specialists. The TA procedure was performed under intratracheal general anesthesia. For all lesions, single or overlapping multiple punctures were performed to cover the index tumor with a margin of 5 mm if possible. MWA or RFA was selected according to the tumor size and location. When TA was completed according to the preablation plan, CEUS was employed to evaluate whether the tumor was ablated completely. Additionally, the operator observed whether there was effusion along the puncture path and whether the contrast agent accumulated in the abdominal cavity.

### 2.6 Surveillance and follow-up

#### 2.6.1 Liver transplantation group

CEUS was performed every day for the first three days after LT. The patient was seen for follow-up visits every week during the first month and every month during the first 6 months. Then, the patient was seen for a follow-up visit every 6 months. Liver function tests, coagulation function tests, abdominal US/CEUS and chest CT were employed at every visit to evaluate the transplanted liver.

#### 2.6.2 Thermal ablation group

US was performed within 72 hours to exclude early complications. Contrast-enhanced CT (CE-CT)/CE-MRI was performed one month after ablation and was taken as the standard reference for the evaluation of technique efficacy. Follow-up was generally required every three to six months. Liver function tests, coagulation function tests, abdominal US or CE-CT/CE-MRI and chest CT were employed at every visit.

#### 2.6.3 Evaluation parameters

For the TA group, complete ablation was defined as complete necrosis of the index tumor confirmed by CE-CT/MRI. Local tumor progression (LTP) was defined as the appearance of tumor recurrence at the edge of the ablation zone after at least one contrast-enhanced image confirmed complete necrosis of the index tumor. For the LT and TA groups, intrahepatic recurrence (IR) was defined as any occurrence of a new nodule of HCC in the liver after at least one contrast-enhanced image confirmed complete necrosis of the index tumor. A major complication was defined as an event that led to substantial morbidity and disability, increased the level of care, lengthened the hospital stay or resulted in hospital admission.

### 2.7 Statistics and analysis

Variables in the two independent groups were compared using a two-sample t test or a Mann−Whitney test for continuous variables and Pearson’s χ2 test or Fisher’s exact test for categorical variables. Cumulative curves of IR and overall survival (OS) curves were estimated by using the Kaplan−Meier method with the log-rank test. Univariate and multivariate analyses of all data were performed using the Cox proportional hazards regression model for IR and binary logistic regression model for major complications.

To minimize the effect of potential confounders on selection bias, propensity scores were generated by using binary logistic regression. Independent variables entered into the propensity model included sex, age, primary or recurrent tumor, PLT count, PT, albumin (ALB), total bilirubin (TB), creatinine (CR), alpha-fetoprotein (AFP), tumor number and maximum tumor size. One-to-two matching between the groups was accomplished by using the caliper matching method.

SPSS 22.0 (SPSS, Chicago, IL, USA) and GraphPad Prism 8.3.1 (GraphPad Software Inc., San Diego, CA, USA) were used for statistical analysis. The *P* value was generated from two-tailed tests. A *P* value less than 0.05 was considered statistically significant.

## 3 Results

### 3.1 Intrahepatic recurrence

**Figure 1:**
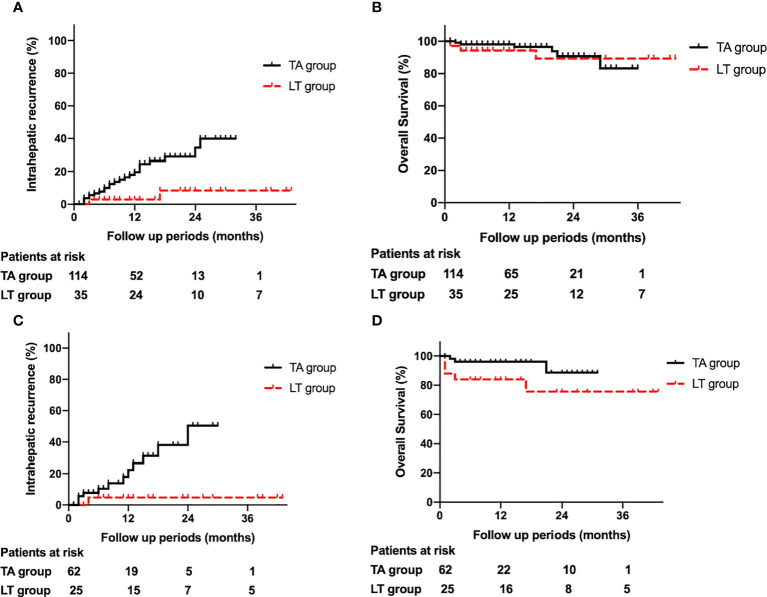
**(A)**. Comparison of intrahepatic recurrence between the thermal ablation and liver transplantation groups. **(B)**. Comparison of overall survival between the thermal ablation and liver transplantation groups. **(C)**. Comparison of intrahepatic recurrence between the thermal ablation and liver transplantation groups after propensity score matching. **(D)**. Comparison of overall survival between the thermal ablation and liver transplantation groups after propensity score matching.

Twenty-one percent (24/114) of patients in the TA group and 5.4% (2/37) of patients in the LT group experienced IR. Among the 24 patients in the TA group with IR, 18 underwent a second TA, 2 underwent surgical resection, and the rest received systematic therapy. Among the 2 patients in the LT group with IR, one underwent transarterial chemoembolism, while the other underwent TA. The 1-, 2-, and 3-year cumulative incidences of IR for the TA group were 21.0%, 32.8%, and 32.8%, respectively, while those for the LT group were 3.0%, 8.4%, and 8.4%, respectively (P=0.025) (**Figure 1A**).

In the TA group, LTP occurred in 3 patients during the follow-up period, yielding an LTP rate of 1.9% (3/157). TA was performed to treat LTP in these three patients. The cumulative incidences of LTP in the ablation group at 1, 2, and 3 years were 2.9%, 2.9%, and 2.9%, respectively.

### 3.2 Overall survival

In the TA group, 6 patients died in the follow-up period and the peri-operative mortality is 0.0% (0/114). By contrast, in the LT group, 5 patients died in the follow-up period and the peri-operative mortality was 8.1% (3/37).

The 1-, 2-, and 3-year cumulative incidences of OS for the TA group were 98.1%, 90.7% and 83.2%, respectively, while those for the LT group were 89.2%, 84.5%, and 84.5%, respectively (P=0.201) (**Figure 1B**).

### 3.3 Major complications

In the TA group, the major complication rate was 4.3% (5/114). No patients died from complications. In the LT group, the major complication rate was 32.4% (12/37), and 5 patients died from complications. The major complication rate between the two groups was significantly different (P<0.001). The details and prognoses of the major complications in the TA and LT groups are listed in **Table 2**.

**Table 2:** Complications of the ablation and transplantation groups before propensity score matching.

Group	Complication	Patient No.	Outcome
TA	Empyema	1	Anti-infectious treatment; catheter drainage; resolved
	Pleural effusion	4	Catheter drainage; resolved
LT	Infection	4	1 patient died from infectious shock; 3 patients were treated with an anti-infectious agent; 2 patients recovered; 1 patient died after one month with pneumonia
	Bleeding	5	2 patients died from hemorrhagic shock in the perioperative period; 2 patients developed toxemia; 1 patient died; 1 patient recovered
	Acute renal failure	1	Hospital admission; case resolved
	Ischemic cholangitis	1	Underwent PTCD and intervention treatments; survived
	Abdominal seeding	1	Radiofrequency ablation; no recurrence; survived

TA, thermal ablation; LT, liver transplantation.

### 3.4 Evaluation of variables affecting major complications and intrahepatic recurrence

In the multivariate analysis (**Table 3**) of all study patients, group (P<0.001; hazard ratio [HR], 0.093; 95% confidence interval [CI]: 0.025, 0.349) was an independent prognostic factor for major complications. For intrahepatic recurrence, group (P=0.015; hazard ratio [HR], 6.842; 95% confidence interval [CI]: 1.453, 32.231) and primary/recurrent tumor (P=0.009; hazard ratio [HR], 0.303; 95% confidence interval [CI]: 0.124, 0.743) were independent prognostic factors.

**Table 3:** Univariant and multivariate analyses of the risk of major complications and intrahepatic recurrence.

Variables	Major complication				Intrahepatic recurrence			
	Univariate analysis		Multivariate analysis		Univariate analysis		Multivariate analysis	
	HR (95% CI)	P	HR (95% CI)	P	HR (95% CI)	P	HR (95% CI)	P
Group	0.096(0.031-0.296)	<0.001	0.107(0.034-0.339)	<0.001	5.296(1.240-22.628)	0.024	6.842(1.453-32.231)	0.015
Sex	0.76(0.161-3.581)	0.729			1.486(0.441-5.008)	0.523		
Age (years)	0.992(0.948-1.038)	0.726			1.047(1.007-1.089)	0.022	1.021(0.981-1.063)	0.313
BMI (≤18.5/>18.5)	0.540(0.107-2.737)	0.457			23.459(0.058-9513.297)	0.303		
Primary/recurrent tumor	2.733(0.976-7.652)	0.056	0.535(0.175-1.632)	0.272	0.351(0.156-0.792)	0.012	0.303(0.124-0.743)	0.009
History of GI bleeding	0.377(0.082-1.733)	0.21			1.230(0.459-3.295)	0.681		
PLT (≤/>50*10^9/L)	0.807(0.247-2.633)	0.722			1.047(0.415-2.645)	0.922		
PT (≤1/>14.5 s)	0.000(0.000-0.000)	0.999			1.962(0261-14.755)	0.513		
ALB* (≤/>35 g/L)	0.699(0.254-1.923)	0.488			1.015(0.451-2.286)	0.971		
TB (≤/>17.1 µmol/L)	0.780(0.270-2.255)	0.647			1.266(0.541-2.964)	0.584		
CR (µmol/L)	1.006(0.987-1.026)	0.512			1.001(0.985-1.017)	0.910		
AFP (≤/>400 ng/ml)	1.143(0.236-5.525)	0.868			23.740(0.073-7705.826)	0.283		
Tumor number	1.432(0.511-4.015)	0.495			0.475(0.213-1.059)	0.069	0.569(0.244-1.327)	0.192
Maximum size (≤/>30 mm)	1.731(0.560-5.346)	0.34			5.746(0.776-42.570)	0.087	2.342(0.292-18.776)	0.423

TA, thermal ablation; LT, liver transplantation; HBV, hepatitis B virus; HCV, hepatitis C virus; GI, gastrointestinal; PLT, platelet; PT, prothrombin time; INR, international normalized ratio; ALB, albumin; TB, total bilirubin; CR, creatinine; AFP, alpha-fetoprotein.

*The distribution of age and ALB satisfied the normal distribution.

### 3.5 Comparison of liver transplantation and thermal ablation after propensity score matching

#### 3.5.1 Baseline characteristics after propensity score matching

The baseline characteristics of the TA and LT groups after propensity score matching are presented in **Table 1**. The baseline characteristics were comparable between the two groups.

#### 3.5.2 Intrahepatic recurrence

In total, 19.4% (12/62) of patients in the TA group and 4.0% (1/25) of patients in the LT group experienced IR. Among the 12 patients in the TA group with IR, 9 underwent TA, 2 underwent surgical resection, and 1 received systematic therapy. The patient in the LT group with IR underwent transarterial chemoembolism. The 1-, 2-, and 3-year cumulative incidences of IR in the TA group were 22.3%, 50.4%, and 50.4%, respectively, while those in the LT group were 4.5%, 4.5%, and 4.5%, respectively (P=0.016) (**Figure 1C**).

In the TA group, LTP occurred in 2 patients during the follow-up period, yielding an LTP rate of 3.2% (2/62). TA was performed to treat LTP in these two patients. The cumulative incidences of LTP in the TA group at 1, 2, and 3 years were 5.8%, 5.8%, and 5.8%, respectively.

#### 3.5.3 Overall survival

In the TA group, 3 patients died in the follow-up period and the peri-operative mortality is 0.0% (0/62). By contrast, in the LT group, 5 patients died in the follow-up period and the peri-operative mortality was 12.0% (3/25).

The 1-, 2-, and 3-year cumulative incidences of OS in the TA group were 96.1%, 88.7%, and 88.7%, respectively, while those in the LT group were 84.6%, 76.2%, and 76.2%, respectively (P=0.07) (**Figure 1D**).

#### 3.5.4 Major complications

In the TA group, the major complication rate was 4.8% (3/62). No patients died from complications. In the LT group, the major complication rate was 36.0% (9/25), and 3 patients died from complications. The major complication rate between the two groups was significantly different (P<0.001). The details and prognoses of major complications in the ablation and transplantation groups are listed in **Table 4** below.

**Table 4:** Complications of the ablation and transplantation groups after propensity score matching.

Group	Complication	Patient No.	Outcome
TA	Empyema	1	Anti-infectious treatment; catheter drainage; resolved
	Pleural effusion	2	Catheter drainage; resolved
LT	Infection	4	1 patient died from infectious shock; 3 patients were treated with an anti-infectious agent and recovered
	Bleeding	3	2 patients died from hemorrhagic shock in the perioperative period; 1 patient developed toxemia and died
	Acute renal failure	1	Hospital admission; resolved
	Ischemia cholangitis	1	Interventional treatment; resolved

TA, thermal ablation; LT, liver transplantation.

## 4 Discussion

According to the results in the present study, the 1-, 2-, and 3-year IR rates were 22.3%, 50.0%, and 50.0% for TA and 4.5%, 4.5%, and 4.5% for LT, respectively, which was generally consistent with the majority of previous reports (13–18). In addition, the 1-, 2-, and 3-year OS rates for TA and LT are also comparable to those in the previous literature (13–18). In contrast, the major complication rates for both groups were slightly higher than that in previously reported data (13–18). The possible reason may be that the presence of CSPH in the enrolled population may increase the risk of major complications, which may be further clarified in future research. Therefore, although the sample size of the present study was relatively small, the consistency of our results with previous reports indicated that the enrolled patients can represent the general population of patients who have undergone LT or TA.

First, the present study revealed that the IR rate in the LT group was significantly lower than that in the TA group, similar to our common opinions. This finding may be explained by the fact that LT removes not only the index tumors but also the microsatellite focus in the diseased liver (19, 20),which may greatly reduce the possibility of IR. In contrast to LT, TA involves inactivation of the tumor in situ, influenced by whether irreversible cell damage occurs in the whole tumor. Nearly one-third of the patients in the TA group experienced IR after the first ablation procedure and underwent a second ablation procedure or transarterial chemoembolism. This influence of treatment selection on the IR rate was also confirmed by multivariate analysis.

Although the IR rate was higher in the TA group than in the LT group, the OS rates of the two groups were statistically comparable. The mortality in the perioperative period was relatively higher in the LT group and mostly related to the incidence of major complications. This could explain why the cumulative 1-year OS rate of the LT group was relatively lower than that of the TA group. After the perioperative period, mortality related to tumor progression seldom occurred. By comparison, the 1-year OS rate of the TA group was as high as 96.1% because the incidence of major complications in the perioperative period was low, and there were no deaths related to major complications. Even if IR occurred, repeated ablation therapy or other treatments still benefited the patients and prolonged the survival time (21). As a result, TA achieved a comparable 1-, 2-, and 3-year OS rate to that with LT.

Regarding the major complications, the incidence rate was significantly lower in the TA group than in the LT group both before and after propensity score matching. In fact, the incidence rate of major complications in the LT group was comparable to that in previous reports (22, 23). In addition, the major complications in the LT group were more severe than those in the TA group (24). The invasiveness of LT could result in a high risk of bleeding in the perioperative period for HCC patients with CSPH, and some of these events are even fatal. Moreover, blood loss may in turn lead to ischemia of the kidney, resulting in acute or chronic kidney failure in the follow-up period (25, 26). Thus, perioperative administration to prevent bleeding is extremely important for LT, especially for patients with CSPH and abnormal coagulation function. By comparison, TA had a lower major complication rate with less severity than LT, which was slightly higher than those previously reported (13, 14, 27, 28), possibly because the enrolled population did have a higher chance of bleeding due to the low PLT count caused by CSPH before treatment. One reason for this finding is the relatively low invasiveness of TA, while the other may be the intraprocedural application of CEUS to monitor puncture path bleeding^12^. If active hemorrhage was confirmed, immediate intervention, such as the administration of hemostasis, was administered. For patients with CSPH, most of whom are prone to hemorrhage during thermal ablation due to hypersplenism or coagulation dysfunction, the application of CEUS has great clinical value. Therefore, TA is a safe choice with a low risk of major complications, which was also confirmed by the results of multivariate analysis.

Apart from the lower incidence rate of complications and a comparable OS rate, the cost of TA is indeed lower than LT, which is one of the reasons why liver transplantation is not suitable for all patients, and also one of the advantages of thermal ablation over liver transplantation.

Portal hypertension is a serious complication of cirrhosis and presents with ascites, hepatic encephalopathy, and bleeding from gastroesophageal varices (3). Portal hypertension is correlated with a poor prognosis in chronic hepatitis patients (11). In clinical practice, measurement of the hepatic venous pressure gradient is the gold standard for the assessment of portal hypertension (29). However, this method is invasive and impractical for repeated follow-up examinations (4). This has led to the noninvasive assessment of portal hypertension, which is identified clinically by the presence of esophageal and gastric varices and/or a PLT count <100*10^9^/L associated with splenomegaly in most previous reports. Thus, the present study employed the condition of CSPH as an inclusion criterion.

There are several limitations to the present study. First, it was retrospective, which may have inevitably caused bias and imbalance between the two groups despite propensity score matching. Second, the sample size of the present study was somewhat small. A larger population may be enrolled in future studies to further validate the current topic.

In conclusion, LT should be recommended as the best option for HCC patients with CSPH. Compared to LT, TA has a lower incidence rate of complications and a higher rate of IR but a comparable OS rate and could be a safe and effective alternative for patients with CSPH.

## Data availability statement

The raw data supporting the conclusions of this article will be made available by the authors, without undue reservation.

## Ethics statement

The studies involving human participants were reviewed and approved by the Institutional Review Board of Third Affiliated Hospital of Sun Yat-sen University. The ethics committee waived the requirement of written informed consent for participation.

## Author contributions

Concept and design: KL and BF; acquisition of data: KL, BF, QZ, EX, and XH; collection of data: YL, and ZY; analysis of data: YL, ZY, ZL, and LY; interpretation of data: YL, ZY, and ZL; manuscript drafting: YL and ZY; revision: KL and BF. All authors contributed to the article and approved the submitted version.
